# Story of the Insane from Year to Year

**Published:** 1907-09-28

**Authors:** 


### STORY OF THE INSANE FROM YEAR TO YEAR.

Eighteenth Annual Series.

### HOSPITAL FOR THE INSANE, NEW NORFOLK, TASMANIA.

The Tasmanian Government recently thought it desirable to appoint a Commission to inspect the general hospitals and asylum of the colony, and Messrs. Elkington, Seager, and Hughes were entrusted to carry out this work. As to the qualifications possessed by these gentlemen, we are not in a position to judge; but we may mention that their report is concise and very well arranged. The portion of it which refers to the general hospitals does not fall into the range of this series, but we are glad to have the opportunity of noticing the section relating to the asylum. At the time of inspection the institution contained 466 patients; and we are told that the condition of the wards and the establishment generally reflected great credit on the medical superintendent and his staff; and the Commissioners add that the patients were treated with great kindness. The Commissioners make several suggestions, and with these we are in general agreement. For instance, it is beyond doubt that the official visitors of a hospital should take no part in the executive; and naturally the Commissioners wish to see such an absurd system removed. It is recommended that the medical superintendent should be directly responsible to the Minister alone, and should have control over the whole staff; indeed, we fail to see how discipline could be properly carried out in the absence of that control. An increase in the staff of attendants is advised; and that the nursing staff should be properly trained is another suggestion. The Commissioners remark on the extraordinary amount of mechanical restraint, and not without reason, for 64 patients had been restrained during the previous year for a total number of 163,222 hours, an amount which is probably greater than for all the asylums in Great Britain put together. Better means of observing newly admitted patients is recommended, and the separation of epileptics from the others should be more efficiently carried out. Here it should be remembered that there may be structural difficulties to overcome before perfect classification could be arrived at, and this is probably the case, as we see that more single-bedded rooms are required. That there should be a separate provision for convicts, and that new kitchens should be built are other suggested improvements.

### THE ROYAL ASYLUM, EDINBURGH.

At the beginning of the year 1906 there were 829 patients in residence. The first admissions reached the high number of 335, and the readmissions 93. The recoveries were 137, the discharged relieved 106, and the deaths 116. On December 31 the number resident had risen to 880, and the average number resident during the year was 870. The recoveries for the year stand at 32 per cent. of the admissions, and the deaths at 13.3 per cent. of the average number resident. The first of these percentages is lower than the County Asylum average and the second is higher. Of the year's deaths 55 were caused by cerebral and spinal diseases, general paralysis accounting for 37 of the total. Phthisis was the cause in 18, pneumonia in 6, and colitis in 4. Various moral causes are assigned as causing the insanity in 15 cases. Among the physical causes hereditary influence comes first with 128, and intemperance in drink follows close with 110. Dr. Clouston's report runs to eleven pages, and there is not a dull paragraph in it as regards style, though much of it may contribute dull feelings to him who reads from the sad nature of the statements. Dr. Clouston shows that in the early sixties it was so uncommon to have a female general paralytic that all the medical staff would go to see the patient; that in 1872 there were no such admissions; that in 1874 there were only 3; and that last year there were 38 female patients admitted with this terrible disease. Further, he points out that 37 of these were rate-paid cases; in fact, there were only 6 general paralytics among the private patients. Drs. Ford Robertson and McCrae devoted all their spare time during the year to the investigation of this disease, and Dr. Clouston tells us that they have, in his opinion, "proved its immediate cause to be a microbe which acts specially on brains that have previously been weakened by dissipation, exhaustion, and poisoning." This is by no means a new idea, but it has not often been so authoritatively stated. Regarding "drink" cases, we find the same tale of increase among women. In the Royal Asylum the percentage has risen from 16.2 to 22, and here again it is rate-paid cases that show the highest proportion. What is our modern system of education doing to lessen this evil habit? We fear next to nothing, and Dr. Clouston is right in saying that "the young at school should surely be taught more about it as a mere branch of knowledge that will help them in their future lives." The asylum during the year was visited by an outbreak of asylum dysentery, from which disease 29 patients suffered, with 4 deaths. The outbreak was in this instance traced to a faulty drain. Dr. Clouston thinks it is quite possible that in future legislation "mental disease may become notifiable, as one means of eliminating the unfit for marriage." Perhaps; but it will be a long time. The cost per head for pauper patients was £35 15s. 4d. per annum, the Craig House cases cost £125 13s. 11d., and the intermediate cases cost £43 19s. 9d., but all these charges would be somewhat reduced by deducting prices of articles sold or supplied to the asylum by the farm. The managers express their sense of obligation to Dr. Clouston for his untiring zeal and unvarying courtesy.

